# From sensation to action: expectation, appraisal, and decision-making in patient-triggered cardiac monitoring

**DOI:** 10.1007/s10865-026-00690-2

**Published:** 2026-07-01

**Authors:** Maeve M. Sargeant, Michael Li, Camden Harrell, Ekin C. Uzunoglu, Steven Mullane, Crystal Miller, Ghanshyam Shantha, Samuel F. Sears

**Affiliations:** 1 Department of Psychology, East Carolina University, Rawl 104, Greenville, NC 27858 USA; 2 Brody School of Medicine, East Carolina University, Greenville, NC USA; 3 Biotronik Inc, Lake Oswego, OR USA; 4 Aiken Regional Medical Centers, Aiken, SC USA; 5 Biotronik NRO, Inc, Lake Oswego, OR USA; 6 Department of Cardiovascular Sciences, East Carolina University, Greenville, NC USA

**Keywords:** Implantable loop recorder, Symptom perception, Signal detection theory, Physical activity, Cardiac physiology

## Abstract

This study examined whether objective physiological arousal predicts patient-triggered recordings (PTRs) in patients with an implantable loop recorder (ILR), to clarify how normative autonomic activation and clinically meaningful rhythm disturbances relate to symptom-triggered monitoring behavior during long-term surveillance. Data were drawn from 2,266 ILR patients enrolled in the CERTITUDE registry between 2019 and 2025; analyses were restricted to the 878 individuals who used the PTR function at least once. Day-level ILR data from the first 12 months following implantation were analyzed. Daily physical activity percent per day (PA), weekly variability in PA, and counts of device-detected atrial fibrillation, bradycardia, and asystole were modeled as predictors of daily PTR counts using within-person repeated-measures correlations and linear mixed-effects models. Subgroup analyses were conducted by primary clinical indication. Daily PA showed near-zero within-person associations with PTR behavior (overall r = − .018), and mixed-effects models yielded very small coefficients. Weekly PA variability was not associated with PTR activation. Device-detected arrhythmic events accounted for only marginal additional variance in PTR counts, and asystole was not associated with PTR use. Patterns were consistent across ILR indications. Overall, objective physiological arousal explained little of the observed variation in PTR behavior. PTR behavior during long-term ILR monitoring appears to be shaped largely by interpretive and decisional processes, rather than by direct detection of physiological events alone. Conceptualizing PTRs as behavioral responses to ambiguity in bodily sensations highlights the importance of patient education, contextualized physiological feedback, and attention to symptom appraisal in the clinical management of ILR patients.

## Introduction

Patients managing cardiovascular conditions are often asked to monitor bodily sensations and respond appropriately to symptoms that may signal clinical risk. This task becomes especially difficult when sensations are intermittent or overlap in subjective presentation, such that similar experiences may reflect benign autonomic fluctuations, physical activity, emotional arousal, or clinically meaningful cardiac events. Deciding when a sensation warrants action, such as seeking reassurance, contacting a clinician, or activating a monitoring tool, represents a form of health behavior: moment-to-moment self-regulation under conditions of uncertainty (Mishel, 1990). Anxiety-related processes may be especially relevant in this context, as anxiety is common in cardiovascular populations and may increase vigilance toward bodily sensations, distress in response to ambiguous cues, and the likelihood that sensations are interpreted as threatening (Celano et al., 2016; Eifert et al., 2000; Du et al., 2023; Sears et al., 2005). Further, heightened attention to bodily sensations and threat-based interpretations have been linked to somatosensory amplification, reassurance-seeking behavior, and activity restriction (Barsky & Wyshak, 1990; Du et al., 2023; Åhlund et al., 2013; Bäck et al., 2020). Understanding what drives symptom-triggered action in cardiovascular populations is central to clarifying how patients navigate uncertainty about ambiguous bodily sensations.

Implantable loop recorders (ILRs) are widely used for long-term rhythm surveillance in patients with atrial fibrillation, syncope, palpitations, cryptogenic stroke, and unexplained falls. In addition to continuously detecting arrhythmic events, ILRs allow patients to manually initiate a recording during perceived symptoms through patient-triggered recordings (PTRs). PTRs are intended to capture rhythm strips during moments patients judge as clinically meaningful, supporting diagnostic clarification and clinical decision-making. However, across studies, PTRs frequently show poor concordance with device-detected arrhythmias, with many patient-initiated recordings occurring in the absence of confirmed rhythm disturbance (Balmelli et al., 2003; Radovanović et al., 2021; Letsas et al., 2025). Although this discordance is well documented, its behavioral meaning remains unclear.

From a health psychology perspective, this mismatch raises a fundamental question: what drives patients to act on bodily sensations during long-term cardiac monitoring? Prior research has often interpreted discordance between PTRs and arrhythmias as a limitation of monitoring yield, device sensitivity, or diagnostic utility (Zimetbaum & Goldman, 2010; Shurrab et al., 2016; Huovinen et al., 2026; Sreeram et al., 2008). Comparatively less attention has been given to PTRs as behavioral responses that reflect how patients perceive, interpret, and appraise internal cues (Neng & Weck, 2015).

The Common-Sense Model (CSM) of self-regulation provides a useful framework for understanding why patients may differ in how they respond to ambiguous sensations. According to the CSM, individuals form illness representations that include beliefs about symptom identity, cause, consequences, and controllability, which guide coping behaviors such as monitoring and reassurance seeking (Leventhal et al., 1980; Hagger & Orbell, 2003). Within this framework, PTRs can be conceptualized as coping behaviors that reflect symptom appraisal. Patients may activate recordings when sensations are interpreted as concerning or diagnostically meaningful, even when the underlying physiological state is benign.

Decision-making under ambiguity further clarifies how symptom-triggered actions may emerge in monitoring contexts. When physiological signals overlap in subjective presentation, actions may reflect the threshold at which sensations are judged as concerning and not the presence of physiological activation alone. Individuals may differ in these decisional thresholds based on prior medical experiences, perceived vulnerability, expectations regarding risk, and tolerance for uncertainty. These factors can bias interpretation toward threat-related meanings and lower the threshold for action in the presence of ambiguous cues (Zacharioudakis et al., 2022).

Two competing explanations are plausible. PTR activation may primarily reflect objective physiological activation (i.e., signal strength), such that increases in autonomic arousal or arrhythmic events reliably drive patient-triggered recordings. Alternatively, PTR behavior may reflect interpretive and decisional processes that determine when ambiguous sensations warrant action. Distinguishing between these explanations is essential for understanding whether PTRs function primarily as physiological detection tools or as behavioral expressions of health decision-making under uncertainty.

Despite the relevance of these processes, prior studies have not systematically evaluated whether objective physiological arousal, both normative and clinically meaningful, predicts PTR behavior across extended monitoring periods. Determining the extent to which objective physiological arousal accounts for PTR behavior is a necessary first step in distinguishing physiological detection from interpretive decision processes. ILRs generate longitudinal data on PA, arrhythmic events, and PTR use, allowing for within-person tests of whether day-to-day variation in objective physiological states aligns with symptom-triggered monitoring behavior over time.

The present study addressed this question by examining whether objective indicators of physiological arousal predict PTR behavior during the first year following ILR implantation. Aim 1 tested whether daily physical activity, representing normative autonomic arousal, was associated with daily PTR counts. Aim 2 examined whether within-person weekly variability in physical activity predicted PTR use. For both aims, we evaluated whether device-detected arrhythmic events (atrial fibrillation, bradycardia, and asystole) accounted for PTR behavior. By testing whether objective physiological arousal accounts for symptom-triggered monitoring behavior, this study directly evaluates whether PTRs function primarily as physiological detection tools or as behavioral expressions of appraisal and decision-making under uncertainty. We hypothesized that associations between objective physiological arousal and PTR activation would be small, consistent with the interpretation that PTR behavior reflects symptom appraisal and decision-making processes in the face of ambiguous bodily sensations rather than direct detection of physiological events alone.

## Methods

This study analyzed data from patients implanted with a BIOMONITOR III or IIIm implantable loop recorder (ILR; BIOTRONIK SE & Co. KG, Berlin, Germany) and enrolled in the CERTITUDE registry. CERTITUDE is a real-world U.S. National dataset linking BIOTRONIK device-derived diagnostic information with Medicare fee-for-service (FFS) inpatient, outpatient, and carrier claims through an executed data use agreement with the Centers for Medicare & Medicaid Services (CMS). The registry protocol was reviewed and approved by the Advarra Institutional Review Board (Columbia, MD), which granted a waiver of informed consent and a full waiver of Health Insurance Portability and Accountability Act authorization.

The present analyses used secondary registry data derived from ILR device transmissions recorded between August 2019 and March 2025. Participants were eligible if they had at least 12 months of available device data and a documented indication for ILR implantation, either as reported to the device manufacturer or identified through ICD-10 diagnosis codes in CMS FFS claims. Indications were not mutually exclusive; however, each patient was assigned a primary clinical indication according to device documentation. The full cohort included 2266 ILR patients/devices, and the primary analytic sample included 878 patients/devices who used the PTR function at least once during the study period.

### Transparency and openness

This study was not preregistered, and the analysis plan was not preregistered. Data for this study are derived from the CERTITUDE registry and are not publicly available due to data use agreements with the Centers for Medicare & Medicaid Services. Researchers interested in potential collaboration or data access procedures would need to contact BIOTRONIK, subject to applicable regulatory, contractual, and institutional approvals. The analytic code was developed and executed by BIOTRONIK as part of proprietary registry analytics and cannot be shared publicly. To support reproducibility, the statistical models, variable definitions, and all inclusion/exclusion criteria are fully described in the manuscript. This report follows the STROBE reporting guidelines for observational studies.

### Study variables

Physical Activity (PA), captured as a percentage of the day, was estimated by the ILR using an internal 3D digital accelerometer that samples motion at 10 Hz. The device algorithm classifies each minute in binary terms (active vs. inactive) based on amplitude thresholds and duration criteria (≥ 30 s of qualifying movement within a 1-minute epoch). Importantly, the BIOMONITOR III and IIIm algorithm integrates cardiac rhythm context when classifying PA, such that activity is only logged if motion patterns coincide with plausible physiological arousal.

PTRs represent patient-initiated device activations, in which the user presses an external handheld activator to capture an electrocardiographic (ECG) recording during a perceived symptomatic event. Each activation generates and stores a cardiac rhythm strip in the device memory and is transmitted via the remote monitoring system directly to their care team. Therefore, PTRs represent patient-initiated responses to perceived cardiac symptoms and not necessarily confirmed arrhythmia events. For this study, PTRs were analyzed as a daily count variable, reflecting the total number of PTRs logged each day.

Additional arrhythmic event variables were also utilized, including episodes of AF, bradycardia, and asystole. ILRs capture these arrhythmic events by continuously recording subcutaneous ECG signals through a single-lead electrode array. Embedded detection algorithms analyze R-R intervals and rhythm morphology to identify arrhythmias based on programmable thresholds for rate, rhythm, and duration. Episodes are stored in device memory and summarized within the remote monitoring system available to clinicians for further interpretation. For this study, arrhythmic episodes were analyzed as a count variable, reflecting the total number of each arrhythmic episode identified per day.

PA and arrhythmias were selected as physiological inputs because they represent two distinct forms of cardiac arousal that patients routinely experience. PA was conceptualized as a marker of normative activity-related autonomic arousal, given that the device’s PA classification incorporates physiological signal context and autonomic activation is a typical and predictable response to physical activity (White & Raven, 2014; Michael et al., 2017). Arrhythmic events were included as markers of clinically meaningful rhythm disturbance. Together, these variables allowed us to examine whether PTR behavior aligned with normative activity-related autonomic arousal, clinically meaningful rhythm disturbance, or processes beyond these measured physiological inputs.

### Statistical analyses

Analyses were restricted to the 878 patients who used the PTR function at least once during the study period (Table 1). All models used ILR data from the first 12 months following implantation. Aim 1 evaluated whether day-to-day device-derived physical activity (PA) was associated with day-to-day variation in PTR counts. We used two complementary within-person approaches: (1) repeated-measures correlations, computed by centering daily PA and PTR values around each individual’s mean to isolate within-person associations; and (2) linear mixed-effects models with random intercepts, treating PTRs as daily count outcomes and PA as a continuous predictor. Subgroup analyses were conducted for each primary ILR indication. For Aim 1, adjusted mixed-effects models included daily counts of device-detected arrhythmic events, including AF, bradycardia, and asystole.

**Table 1:** Demographics

Baseline characteristics	
Age (years), Mean ± SD	74.9 ± 7.7
Follow-up period (years), Mean ± SD	2.5 ± 1.3
Gender, n (%)	
Male	423 (48.2%)
Female	455 (51.8%)
Ethnicity n (%)	
Caucasian	798 (90.9%)
Other	80 (9.1%)

Aim 2 evaluated whether within-week variability in PA was associated with PTR behavior. PA variability was defined as the within-week standard deviation of daily PA%. Repeated-measures correlations and mixed-effects models were again estimated, with weekly PA variability as the predictor. For Aim 2, adjusted mixed-effects models included device-detected arrhythmic events and monitoring week to account for changes in PTR use over the monitoring period. Age and sex were summarized descriptively but were not included as primary covariates because the primary analyses focused on within-person alignment between repeated device-derived physiological inputs and PTR activation, rather than between-person prediction of PTR use. Primary ILR indication was addressed through indication-specific subgroup analyses. Full mixed-effects model results are reported in Table 2. Statistical significance was defined as *p* < .05; effect sizes were interpreted with attention to their clinical relevance given the large sample size.

**Table 2:** Mixed-effects model results for daily device-derived predictors of patient-triggered recording activation

Cohort	Parameter	Estimate	SE	95% CI	*p*
Overall	Intercept	0.07665	0.00843	0.06011, 0.09319	< 0.0001
	Physical activity	− 0.00155	0.00047	− 0.00247, − 0.00064	0.0009
	Bradycardia	0.00044	0.00013	0.00018, 0.0007	0.0008
	Atrial fibrillation	0.0019	0.00075	0.00044, 0.00336	0.0110
	Asystole	− 0.0001	0.00006	− 0.00021, 0.00002	0.0939
AF management	Intercept	0.07377	0.01536	0.04357, 0.104	< 0.0001
	Physical activity	− 0.00142	0.00081	− 0.003, 0.00016	0.0783
	Bradycardia	0.00048	0.00022	0.00004, 0.00092	0.0325
	Atrial fibrillation	0.00403	0.00144	0.0012, 0.00685	0.0053
	Asystole	− 0.00006	0.00008	− 0.00022, 0.0001	0.4798
Cryptogenic stroke	Intercept	0.04485	0.00748	0.02999, 0.05971	< 0.0001
	Physical activity	− 0.00183	0.00054	− 0.00289, − 0.00078	0.0007
	Bradycardia	0.00049	0.00015	0.00018, 0.00079	0.0016
	Atrial fibrillation	− 0.00013	0.00042	− 0.00095, 0.00068	0.7499
	Asystole	0.00008	0.00019	− 0.0003, 0.00047	0.6744
Unexplained syncope	Intercept	0.07783	0.01698	0.04436, 0.1113	< 0.0001
	Physical activity	− 0.00011	0.001	− 0.00206, 0.00184	0.9116
	Bradycardia	0.00077	0.0003	0.00018, 0.00136	0.0107
	Atrial fibrillation	0.00277	0.00217	− 0.00149, 0.00702	0.2023
	Asystole	− 0.00078	0.0003	− 0.00137, − 0.00019	0.0099
Unexplained fall	Intercept	0.06469	0.02542	0.01198, 0.1174	0.0184
	Physical activity	− 0.00063	0.00158	− 0.00373, 0.00246	0.6872
	Bradycardia	− 0.00499	0.00087	− 0.0067, − 0.00328	< 0.0001
	Atrial fibrillation	0.00913	0.00527	− 0.00121, 0.01946	0.0834
	Asystole	− 0.00095	0.00223	− 0.00533, 0.00343	0.6692
Palpitations	Intercept	0.09203	0.01615	0.06015, 0.1239	< 0.0001
	Physical activity	− 0.00287	0.00095	− 0.00473, − 0.001	0.0026
	Bradycardia	0.00027	0.00025	− 0.00022, 0.00077	0.2807
	Atrial fibrillation	0.0023	0.00249	− 0.00259, 0.00718	0.3573
	Asystole	− 0.00008	0.0001	− 0.00029, 0.00012	0.4244

Estimates are unstandardized fixed-effect coefficients. The model included daily physical activity and device-detected arrhythmic events, including atrial fibrillation, bradycardia, and asystole. Primary clinical indication was evaluated through indication-specific models. PTR = patient-triggered recording; CI = confidence interval

## Results

The mean age of the cohort was 75.5 ± 7.3 years, with 50.4% male, and mean implantation duration of 2.5 ± 1.3 years. Primary indication for ILR implantation was diverse: 1047 patients for atrial fibrillation (AF) management (mean age: 75.3 ± 6.6 years; 57.4% male), 452 for unexplained syncope (76.1 ± 8.3 years; 47.6% male), 332 for cryptogenic stroke (75.4 ± 7.2 years; 41.9% male), 395 for palpitations (75.5 ± 7.6 years; 43.3% male), and 40 for unexplained falls (75.4 ± 8.0 years; 37.5% male). Overall, 878 patients (38.7%) used the PTR function at least once during the study period. The prevalence of PTR use varied by indication: 35.8% in AF management, 46.0% in unexplained syncope, 28.3% in cryptogenic stroke, 45.1% in palpitations, and 57.5% in unexplained falls. Among patients who engaged the PTR function, the mean age was 74.9 ± 7.7 years, with 48.2% male, and mean follow-up duration of 2.5 ± 1.3 years.

### Aim 1: daily physical activity and PTRs

Within-person repeated-measures correlations between centered daily PA and centered daily PTR counts were negligible in magnitude for the overall sample (*r* = − .018, *p* = .0002) and across all indication groups, including AF management (*r* = − .014, *p* = .07), cryptogenic stroke (*r* = − .048, *p* = .001), unexplained syncope (*r* = − .003, *p* = .79), unexplained falls (*r* = − .023, *p* = .43), and palpitations (*r* = − .041, *p* < .001). Although some correlations reached statistical significance, all effect sizes were small, consistent with minimal within-person alignment between daily PA% and PTR activation.

Linear mixed-effects models evaluating daily PA in relation to daily PTR counts produced small negative coefficients in the overall sample (estimate = − 0.0016, SE = 0.00046, *p* = .0008). Interpreted on the observed PA scale (%/day), this coefficient corresponds to an expected change of − 0.0016 PTRs per 1%-point (pp) increase in PA% (e.g. − 0.016 PTRs per 10 pp increase), indicating a negligible difference in practical terms. Indication-specific models yielded non-significant results for AF management (*p* = .07), syncope (*p* = .85), and falls (*p* = .98). Statistically significant coefficients of small magnitude were observed for cryptogenic stroke (estimate = − 0.00168, SE = 0.00052, *p* = .0013) and palpitations (estimate = − 0.00291, SE = 0.00095, *p* = .0023).

In models adjusting for device-detected arrhythmic events, the association between daily PA and PTRs in the overall sample remained statistically significant (estimate = − 0.00155, SE = 0.00047, *p* = .0009). Bradycardia (estimate = 0.00044, SE = 0.00013, *p* = .0008) and AF episodes (estimate = 0.00190, SE = 0.00075, *p* = .011) were associated with higher PTR counts, whereas asystole was not (*p* = .094). Indication-specific adjusted models showed similar patterns, with small statistically significant coefficients for PA in cryptogenic stroke and palpitations, and non-significant associations in other groups.

### Aim 2: weekly physical activity variability and PTRs

Repeated-measures correlations between centered weekly PA variability (defined as within-week standard deviation of daily PA%) and centered PTR counts were small and non-significant in the overall sample (*r* = − .0066, *p* = .17) and across indication groups (all |r| < 0.03, all *p* > .16). Mixed-effects models using weekly PA variability as the predictor did not yield significant associations in the overall sample (estimate = − 0.00111, SE = 0.00100, *p* = .27) or within any indication group (all *p* > .10). In adjusted mixed-effects models including arrhythmic events and time (week), the association between PA variability and PTRs in the overall sample approached but did not reach statistical significance (estimate = − 0.00194, SE = 0.00100, *p* = .053). Bradycardia and AF were associated with higher PTR counts (*p* = .005 and *p* = .042, respectively), whereas asystole was not (*p* = .089). Time was significantly associated with PTR counts in the overall sample (estimate = − 0.00203, SE = 0.00008, *p* < .001), indicating a progressive decline in patient-triggered recordings over time, with similar patterns observed across indication groups.

## Discussion

The present study examined whether objective physiological arousal is associated with when patients activate PTRs during long-term cardiovascular monitoring. Across all analytic approaches, associations between PA and PTR behavior were near zero, both at the daily level and in terms of week-to-week variability. Device-detected arrhythmic events were statistically associated with PTRs in adjusted models, but effect sizes were small, and asystole was not associated with PTR activation. Together, these findings suggest that objective physiological arousal, whether normative autonomic activation or clinically meaningful rhythm disturbance, accounts for very little of the observed variation in PTR behavior. Because objective physiological indicators accounted for little day-to-day variation in PTR use, the decision to activate the device likely involves factors beyond physiological activation alone. To interpret these processes, we draw on three complementary theoretical frameworks, Signal Detection Theory (SDT), the Common-Sense Model (CSM) of self-regulation, and interoceptive inference.

## PTRs as decisions under uncertainty

SDT provides a foundation for understanding PTR behaviors as decisions made under conditions of uncertainty. This framework distinguishes sensory evidence (“signal”) from background variability (“noise”) (Green & Swets, 1966; Macmillan & Creelman, 2005). For cardiac patients, clinically meaningful physiological symptoms, such as atrial fibrillation or bradycardia, constitute the “signal”, whereas physiological symptoms of normative autonomic fluctuations constitute “noise”. Because these sources of arousal can produce overlapping sensations, the sensory evidence available to the patient often does not cleanly distinguish signal from noise. SDT further suggests that when signal and noise distributions overlap substantially, behavioral responses will be determined less by signal strength and more by the individual’s decision criterion: the amount of internal evidence required before acting (Green & Swets, 1966; Macmillan & Creelman, 2005). When the criterion is low, individuals act upon weaker or more ambiguous signals, increasing “hits” but also increasing false alarms; when the criterion is high, false alarms decrease, but misses of true signals increase. Within an SDT framework, the central implication of our findings is that variability in PTR behavior is better explained by differences in response criteria than by differences in physiological input. Daily PA, a reliable source of objective autonomic arousal, showed near-zero within-person associations with PTRs, and documented arrhythmic events accounted for only minimal additional variance. If PTR activation were driven primarily by physiological signal strength, stronger within-person coupling would be expected. Instead, the observed pattern is more consistent with a criterion-based account, in which patients differ in how much internal evidence they require before initiating PTRs.

### Why criteria differ: illness beliefs and symptom monitoring

While the SDT framework specifies *how* decisions are made under uncertainty, the Common-Sense Model (CSM) of self-regulation helps explain *why* decision thresholds differ across patients. The CSM proposes that bodily sensations are interpreted through illness-related beliefs about symptom identity, perceived cause, expected consequences, and controllability (Leventhal et al., 1980). Importantly, these beliefs vary widely across individuals based on prior medical experiences, diagnostic histories, and interactions with healthcare systems. These belief differences shape how ambiguous physiological sensations are evaluated (Hagger & Orbell, 2003). For some patients, similar sensations may be interpreted as benign, expected, or manageable, such that a higher level of internal evidence is required before the decision criterion for action is reached. For others, the same sensations may be interpreted as signals of potential danger or loss of control, bringing sensations closer to the decision criterion and increasing the probability of action in the presence of ambiguous cues. Cognitive-affective factors such as cardiac anxiety, intolerance of uncertainty, and perceived control may further bias these appraisals by increasing the perceived cost of missed events or increasing the likelihood that ambiguous sensations are interpreted as clinically meaningful (Adams et al., 2022; Heathcote et al., 2021; Bredemeier et al., 2023).

Within this framework, PTRs may be understood as behavioral expressions of symptom appraisal rather than direct responses to physiological disturbance. Patients activate the device not simply when sensations are strong, but when sensations are interpreted as relevant, threatening, or diagnostically meaningful. This helps explain why PTR behavior shows some alignment with objective physiological arousal, albeit weakly, yet maintains substantial variability across patients. This interpretation also helps explain why PTR behavior can persist despite repeated benign findings: surveillance contexts may reinforce vigilance-oriented coping even when objective evidence does not indicate acute pathology.

### Interoceptive inference and the role of expectations

Interoceptive inference complements the CSM by specifying how illness-related beliefs can influence perception when sensory input is uncertain (Seth & Friston, 2016). This framework suggests that perception reflects an integration of sensory information with prior expectations (“priors”), with greater reliance on priors when sensory evidence is noisy or ambiguous (Seth & Friston, 2016; Barrett & Simmons, 2015). In the context of cardiac sensations, where similar physiological sensations could be benign or dangerous, patients may rely more heavily on expectations about vulnerability, prior episodes, and threat-related interpretations (e.g., expectations of arrhythmia, beliefs about vulnerability). This can bias perception toward interpreting ambiguous arousal as clinically meaningful, increasing the subjective weight of sensory evidence that is then evaluated against the decision criterion described by SDT (Seth & Friston, 2016). This framework helps explain why PA, despite reliably producing physiological arousal, showed minimal association with PTR behavior: autonomic activity alone may not prompt threat-related interpretations in the absence of expectancy-based priors.

### Integration of frameworks

**Fig. 1:**
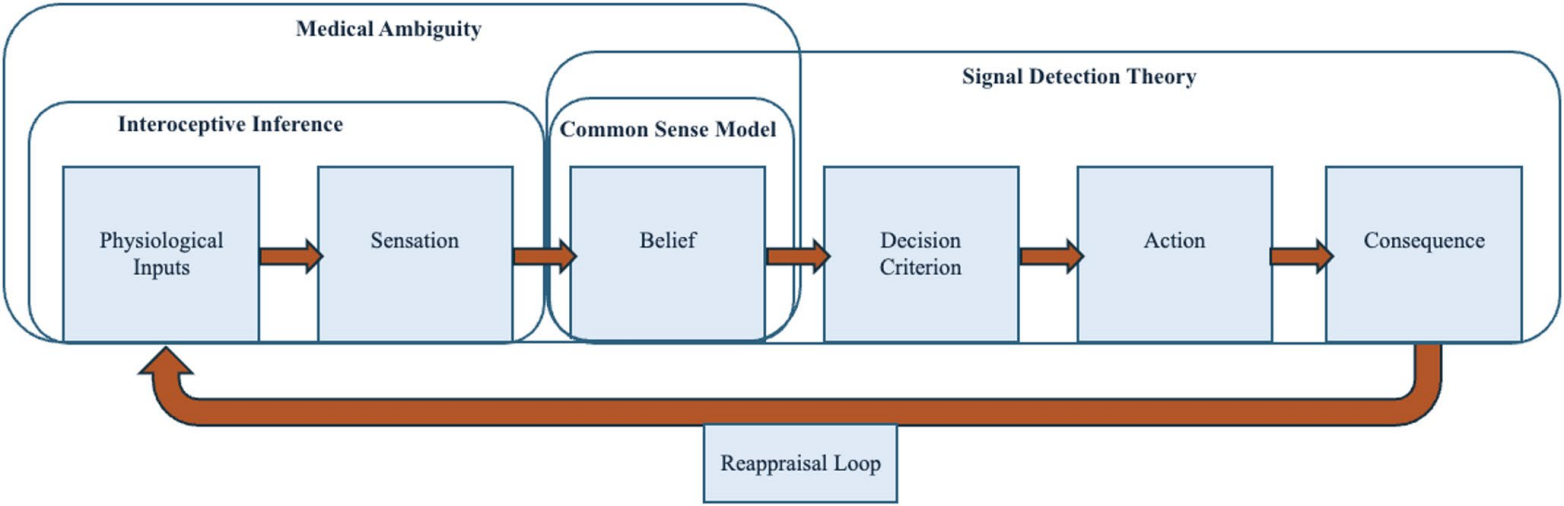
Theoretical conceptualization map

Figure 1 illustrates the integration of these frameworks. Physiological inputs such as PA and arrhythmias generate sensations that are often ambiguous. These sensations are interpreted through prior expectations (interoceptive inference) and illness-related beliefs (CSM), which together shape the decision criterion described by SDT. PTR activation represents the behavioral output of this appraisal and decision process. The subsequent consequence, such as clinical feedback or typically the absence thereof, creates a reappraisal loop that updates prior beliefs and adjusts future decision thresholds. This integrated model highlights how sensation, interpretation, and decision-making may interact to produce the observed misalignment between physiology and PTR behavior. Further, PTR behavior may also evolve over time via a dynamic reappraisal loop. Within the CSM, coping actions are followed by feedback, which updates beliefs and recalibrates thresholds (Leventhal et al., 1980). For ILR patients, each PTR can yield objective rhythm information and communication from their monitoring clinician. Communicated normal rhythm findings may raise the threshold for future activations, whereas confirmed arrhythmias may lower it. The downward trend in PTR use across weeks observed in adjusted models may reflect this recalibration process. Future work is needed to model these dynamics more explicitly.

### Clinical implications

Patient-triggered recordings appear to mark moments when internal sensations exceeded a personally salient threshold, probably reflecting uncertainty, fear, or loss of perceived control, rather than moments when objective physiology necessarily crossed a pathological boundary. Accordingly, the primary clinical implication of these findings is not that PTRs are uninformative or should be discounted, but that they convey a different kind of clinically meaningful information than arrhythmia detection alone. From this perspective, PTRs represent patient-initiated communication about symptom appraisal rather than direct indicators of physiological abnormality.

Accordingly, the clinical task is not to eliminate this form of patient communication, but to support adaptive calibration of the decision criterion over time through consistent, interpretable feedback about benign versus concerning symptom patterns. Importantly, SDT does not define a single ‘correct’ threshold; decision criteria depend on perceived costs of misses versus false alarms (Green & Swets, 1966; Macmillan & Creelman, 2005). In cardiac populations, thresholds are often understandably biased toward sensitivity, particularly early in monitoring or following salient or distressing physiological experiences such as ICD shocks or unexplained symptomatic episodes (Attanasio et al., 2015; Sears et al., 2005, 2011; Ladwig et al., 2008). From a decision-theoretic perspective, such shifts are consistent with adaptive criterion adjustment under heightened unexpected uncertainty, where the perceived cost of “misses outweighs the cost of false alarms” (Payzan-LeNestour & Bossaerts, 2011). Rather than viewing this sensitivity as maladaptive, it may be more appropriate to understand it as a protective response that can be gradually refined as patients accumulate experience and feedback.

These findings suggest a potential opportunity for a multidisciplinary, compassion-centered model of care, in which different members of the healthcare team contribute complementary but coordinated roles. Cardiac device teams and monitoring clinicians are uniquely positioned to provide timely, concrete feedback that contextualizes PTR events. When benign findings are communicated alongside brief explanations of expected autonomic sensations (e.g., exertion-related heart rate changes, positional effects, or normative arousal), PTR reviews can become opportunities for structured interpretation of sensations in relation to objective findings, rather than instances of reassurance alone. Importantly, such feedback may benefit from explicitly normalizing the patient’s response, acknowledging that activating the device in the face of ambiguous sensations is understandable and reasonable, while also clarifying what the available data suggest about safety and risk. In this way, PTR review functions not only as event adjudication, but as guided sense-making that supports learning and recalibration over time.

When clinically indicated, behavioral health providers may address the psychological processes that shape how sensations are interpreted and acted upon. CBT-informed approaches have been used to help patients identify and evaluate threat-based appraisals of bodily sensations (e.g., catastrophizing or overestimation of risk) and to consider alternative interpretations, as described in work with similar cardiovascular populations (Draheim & Anderson, 2021; Johnsson et al., 2025; Irvine et al., 2011). ACT-informed approaches have similarly been applied to support patients in noticing internal sensations without immediate threat-based responding, clarifying valued life directions (such as maintaining PA), and choosing flexible actions even in the presence of uncertainty (Grimaldi et al., 2025). Across these frameworks, structured engagement with benign physiological sensations and graded activity practice, implemented in collaboration with the medical team, represent potential strategies for targeting the learned association between bodily sensations and danger. By increasing tolerance for benign arousal and supporting continued functional engagement, such approaches align closely with the interpretive and decisional processes suggested by the present findings.

Compassion-Focused Theory (CFT) offers a complementary framework for this work. From a CFT perspective, heightened vigilance and low decision thresholds are not errors to be corrected, but expressions of a threat-protection system operating under conditions of perceived vulnerability (Gilbert, 2009). Framing PTR use as an understandable attempt to seek safety may help reduce self-critical or defensive responses that can interfere with learning and recalibration over time. Across disciplines, a shared compassionate stance that validates the patient’s intent (“your body felt concerning, and it makes sense that you checked”) while gently supporting recalibration (“and here’s what this signal usually means”) may facilitate movement from threat-driven monitoring toward more informed and flexible self-regulation. Importantly, such an approach supports recalibration without minimizing patient experience, allowing patients to adjust decision thresholds without feeling dismissed or unheard.

These implications are especially important given evidence that ILR patients engage in markedly low levels of PA (Sargeant et al., 2024). If benign exertion-related sensations are repeatedly interpreted as dangerous, symptom-triggered monitoring may inadvertently reinforce inactivity and functional restriction. A coordinated approach that combines compassionate normalization, clear physiological education, and consistent device feedback, alongside behavioral interventions that build tolerance for benign arousal, may help protect both medical safety and quality of life. In this way, PTRs are not signals to be ignored, but invitations for collaborative care that integrates physiology, psychology, and lived experience.

### Strengths, limitations, and future directions

This study has several strengths, including a large and clinically diverse national cohort, extended monitoring duration, and integration of objective physiological and behavioral data derived from ILRs. Leveraging an average of 2.5 years of daily data from 878 patients allowed for highly powered within-person tests of whether objective indicators of physiological arousal systematically align with PTR behavior across long-term surveillance. The analytic sample consisted primarily of older U.S. adults enrolled in Medicare fee-for-service who were undergoing implantable loop recorder monitoring for established clinical indications. This demographic profile reflects the epidemiology of arrhythmic and cerebrovascular conditions in which ILRs are most frequently deployed and therefore represents a clinically appropriate population for examining patient-triggered activation behavior in real-world surveillance contexts.

Several limitations should be noted. First, PTR activation is a momentary behavioral decision, whereas the physiological predictors examined in this study were aggregated at the day level; as such, the present analyses do not assess whether physiological changes occur immediately proximal to individual PTR events. Rather, they examine whether days characterized by greater cumulative physiological arousal, indexed by both normative autonomic activation (physical activity) and clinically meaningful rhythm disturbances, are also days with more frequent PTR activation. If PTR behavior were primarily driven by physiological arousal alone, such alignment would be expected to emerge even at this broader temporal scale. This temporal scale reflects a structural feature of real-world implantable monitoring: current ILR systems do not retain continuous high-resolution physiological data across multi-year surveillance periods due to data storage and transmission constraints.

An additional consideration is that analyses were restricted to patients who activated the PTR function at least once during the monitoring period. Patients who did not use PTR may differ from users in ways not captured in registry data, including variation in device education, familiarity with the activator, perceived reliance on automatic arrhythmia detection, clinician messaging regarding appropriate activation, or symptom interpretation. Because these contextual factors are not available in registry datasets, their influence on PTR engagement and activation patterns cannot be directly evaluated in the present study. Additionally, the CERTITUDE registry did not include validated measures of general anxiety, cardiac anxiety, interoceptive sensitivity, intolerance of uncertainty, perceived control, or real-time symptom appraisal; therefore, these proposed appraisal-related mechanisms could not be directly tested in the present analyses. Finally, medication use, functional status, and mobility impairment were also not available in the present analytic dataset. These factors may influence device-derived PA%, either by altering activity capacity, limiting movement, or modifying physiological responses to activity. Future studies that integrate medication data and functional or mobility assessments may help clarify how patient-level physical capacity and treatment factors shape activity-related physiological activation and symptom-triggered monitoring behavior.

Future research could incorporate formal Signal Detection Theory metrics to estimate individual decision thresholds and examine how these thresholds shift over time. Integrating patient-reported symptom characteristics, cardiac anxiety, uncertainty tolerance, and perceived control would allow for direct testing of moderators hypothesized by the proposed theoretical frameworks. Dynamic models using higher-resolution physiological data could further clarify how physiological signals, expectations, and appraisal processes interact at shorter timescales to shape PTR decision-making.

## Conclusion

The minimal alignment between objective physiological indicators and PTR activation suggests that symptom-triggered monitoring cannot be fully explained by device-captured cardiac physiology alone. An integrated framework drawing on Signal Detection Theory, the Common-Sense Model, and interoceptive inference provides a cohesive explanation for this dissociation by emphasizing expectations, appraisal, and criterion-based decision-making processes in the choice to activate PTRs. Consistent with this view, observed associations between PA% and PTR behavior were negligible in magnitude despite statistical significance in some analyses, underscoring the importance of effect size and interpretability in large datasets. Together, these findings suggest that PTRs should be interpreted not only as potential markers of rhythm disturbance, but also as patient-initiated indicators of perceived symptoms, uncertainty, and the decision to seek clarification. Clinically, this supports a coordinated, team-based approach in which device clinics provide timely physiological interpretation and normalization, nursing reinforces education and self-management strategies, and behavioral health may address fear, avoidance, and tolerance of benign arousal when these processes contribute to distress or functional restriction.
